# Establishing a 3D Spheroid Model of Cholinergic Neurons from SH-SY5Y Cells for Neurotoxicity Assessment

**DOI:** 10.3390/toxins17070336

**Published:** 2025-07-02

**Authors:** Felipe Franco-Campos, Mónica Fernández-Franzón, Yelko Rodríguez-Carrasco, María-José Ruiz

**Affiliations:** 1 Research Group in Alternative Methods for Determining Toxics Effects and Risk Assessment of Contaminants and Mixtures (RiskTox), Universitat de València, Av. Vicent Andrés Estellés s/n, 46100 Burjassot, Spain; felipe.franco@uv.es (F.F.-C.); monica.fernandez@uv.es (M.F.-F.); m.jose.ruiz@uv.es (M.-J.R.); 2 Laboratory of Food Chemistry and Toxicology, Faculty of Pharmacy, Universitat de València, Av. Vicent Andrés Estellés s/n, 46100 Burjassot, Spain

**Keywords:** spheroids, SH-SY5Y, differentiation, cholinergic neurons, neurotoxicity

## Abstract

The nervous system maintains homeostasis and coordinated behavior through complex neuronal and glial cells. Traditional models, such as primary rodent neurons and human-induced pluripotent stem cell (hIPSC)-derived neurons, have advanced our understanding of neuronal function and neurotoxic damage; however, they are costly and labor-intensive. SH-SY5Y cells, an immortalized human neuroblastoma cell line, provide a more accessible alternative for studying neuronal processes and neurotoxicity. However, their limited capacity to differentiate into specific neuronal phenotypes remains a challenge. To address this limitation, differentiation protocols using neuronal factors and vitamins have been developed, primarily in two-dimensional (2D) cultures, which reduces physiological relevance. Here, we present a novel three-dimensional (3D) SH-SY5Y model incorporating 2D differentiation protocols to generate cholinergic neurons (ChAT+). This model enhances neurotoxicity studies related to pesticides and mycotoxins. Our protocol produces homogeneous spheroids differentiated into cholinergic neurons using serum restriction and specific factors, maintaining viability and circularity for up to 22 days. Differentiation was validated by immunofluorescence and Western blot by Choline acetyltransferase (ChAT) expression. This scalable and reproducible 3D model provides a valuable in vitro tool for neurotoxicological research, improving physiological relevance and enabling the study of cholinergic neuron differentiation and function.

## 1. Introduction

The nervous system (NS) is a highly intricate structure crucial for maintaining homeostasis and enabling coordinated behavior. The NS is recognized as the most complex structure known in biology, with neurons serving as the principal cells responsible for receiving internal and external stimuli, processing this information, and initiating responses through electrical and chemical signaling supported by glial cells [1,2]. Historically, the advancement of knowledge concerning the NS has depended on the availability of research tools, which have facilitated the development of techniques that improve our comprehension of this system [3].

In vitro models are widely used in neuroscience and toxicology due to their cost-effectiveness, reduced ethical concerns compared to in vivo approaches, and the ability to precisely control the cellular microenvironment and experimental conditions, which facilitates reproducibility and mechanistic studies. Currently, cell cultures represent one of the principal sources of information, enabling the characterization of diseases, lesions, and the toxicological effects of various agents despite the inherent complexity of the NS [4]. Recent studies have used primary rodent neurons, human-induced pluripotent stem cells (hIPSC), or neuronal cancer cell lines such as LUHMES to study the complexity of the NS [5,6]. Although powerful, these approaches are often labor-intensive. Consequently, there is significant interest in developing a cost-effective and reproducible cell model that can be easily scaled to enhance for repetition and reproducibility. This context has spurred the ongoing innovation of in vitro alternative methods to animal testing, thereby supporting the 3Rs strategy (reduction, refinement, and replacement).

SH-SY5Y neuroblastoma cells are one of the most commonly used human cells lines in neurobiology. Under standard culture conditions, these cells can proliferate rapidly and show an epithelial-like morphology predominating over neuronal cells [7]. Several protocols have been established in 2D cultures developed to induce differentiation favoring neuronal cells over epithelial morphology into various neuronal phenotypes depending on requirements, including dopaminergic and cholinergic neurons [8,9]. This differentiation is achieved by applying specific growth factors, molecules, and conditions that simulate the processes involved in neuronal migration during development in vivo. Retinoic acid (RA) is a potent morphogen that has been shown to promote the differentiation of SH-SY5Y cells into a neuronal-like phenotype characterized by the extension of neurites and the expression of various neuronal markers [10,11]. The addition of brain-derived neurotrophic factor (BDNF) into RA-treated SH-SY5Y cells has been shown to further enhance the expression of important proteins involved in the functionality of cholinergic neurons, which can serve as cholinergic markers, such as choline acetyltransferase (ChAT) and acetylcholinesterase (AChE), one of the principals indicating a shift towards a cholinergic neuronal fate [12,13].

However, all protocols to differentiate SH-SY5Y cells are performed in traditional 2D formats, often failing to capture the intricate cell–cell and cell–matrix interactions seen in vivo. This limitation has sparked interest in 3D cell culture models, which provide a more physiologically relevant environment [14,15]. Spheroids, a common 3D model, allow for better replication in vivo cellular organization, gene expression, and metabolic activities. By elating the complex interactions in living tissues, 3D cultures offer a more accurate platform for studying drug penetration, toxicity, and efficacy, leading to insights that are more translatable to human physiology.

This study aimed to develop a rapid and accessible 3D model of SH-SY5Y cells and to integrate it with 2D differentiated protocols to obtain a cholinergic phenotype (ChAT+) in these spheroids. This novel SH-SY5Y spheroid (ChAT+) model is more representative of in vivo conditions and can be utilized in specific research domains. It can facilitate studies of neurotoxic effects and damage caused by pesticides, mycotoxins, or other neurotoxic compounds.

## 2. Results

### 2.1. Differentiation Protocol and Tracking of Differentiated Spheroids

**Figure 1:**
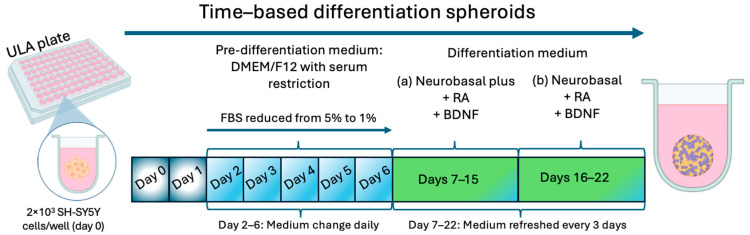
Schematic representation of the spheroid differentiation protocol. Spheroids were grown in 96-well round-bottom ultra-low-attachment (ULA) plates for a duration of 22 days. Each plate was used to generate a single spheroid per well. The differentiation medium (**a**) is as follows: it comprises Neurobasal Plus medium, 10 µM RA and 50 ng/mL BDNF. The differentiation medium (**b**) comprises Neurobasal medium with 10 µM RA and 50 ng/mL BDNF.

Following a review of the protocol currently in use in our laboratory and a comparison with the existing literature on the differentiation of 2D SH-SY5Y cells, we decided to implement a 22-day protocol incorporating AR, BDNF, and serum restriction as the primary variables. Figure 1 illustrates the progression of the protocol, which was initiated with a seeded density of 2000 cells per well on day 0. It also depicts the days of serum restriction and the various culture media employed to obtain SH-SY5Y spheroids differentiated into a cholinergic phenotype (ChAT+).

**Figure 2:**
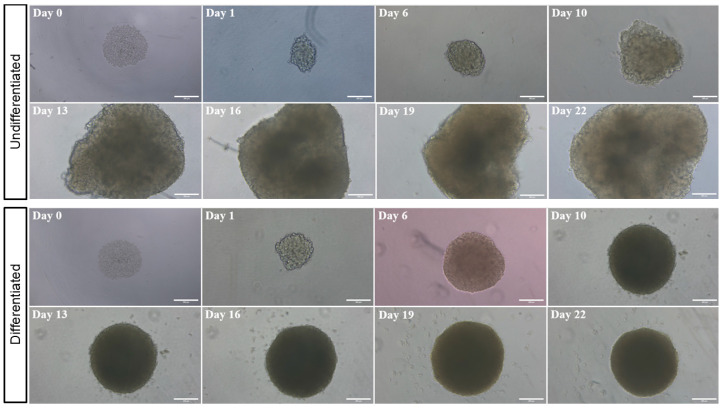
Tracking of the differentiation and undifferentiated status of SH-SY5Y spheroids over a 22-day growth period using ultra-low-attachment (ULA) 96-well round-bottomed plates. The undifferentiated spheroids were maintained for a period of 22 days, during which time the DMEM/F12 medium was refreshed every 3 days. The differentiated spheroids were incubated for 22 days in different culture media. A single spheroid was formed in each well of the culture plate. From day 0 onwards, images were captured at regular intervals throughout the course of the protocol. Imaging was conducted using a Zeiss Axio Observer optical microscope (Zeiss Microscopy) at 10× magnification. Scale bars represent 200 μm.

The undifferentiated SH-SY5Y spheroids exhibited a rapid and disorganized growth pattern, with a progressive deterioration of their spheroidal morphology after day 6 (Figure 2). Additionally, a sphericity index (SI) index below 0.9 was observed from day 10 onward. By day 22, the spheroids had completely lost their circularity, which suggests uncontrolled growth. By contrast, the differentiated spheroids demonstrated a well-defined SI index and maintained their spheroidal morphology throughout the study period (Figure 2). From the outset of the culture process until day 22, the spheroids retained their spherical shape, indicating that the controlled growth process was being successfully completed.

### 2.2. ChAT and MAP2 Protein Expression on Differentiated Spheroids

**Figure 3:**
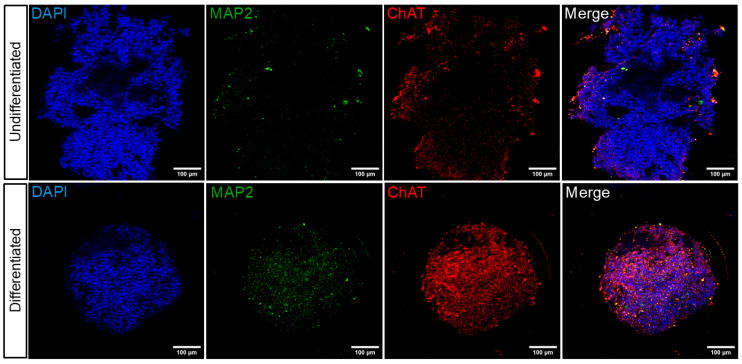
Expression of ChAT and MAP2 in differentiated SH-SY5Y spheroids. The images show the results of the indirect immunofluorescence against ChAT and MAP2 markers in SH-SY5Y undifferentiated and differentiated spheroids. The nucleus was stained with DAPI, resulting in blue coloration. Subsequently, the spheroids were photographed at 20× magnification, with scale bars measuring 100 μm.

The differentiation of spheroids into a cholinergic phenotype was evaluated using indirect immunofluorescence according to the established differentiation protocol. As illustrated in Figure 3, differentiated spheroids exhibited a positive and homogeneous signal for MAP2 and ChAT markers compared to undifferentiated spheroids, which exhibit a low signal only in areas where cells are concentrated and without spheroid morphology.

### 2.3. Effect of Differentiation Spheroids on Cell Cycle and ChAT Expression by Western Blot

**Figure 4:**
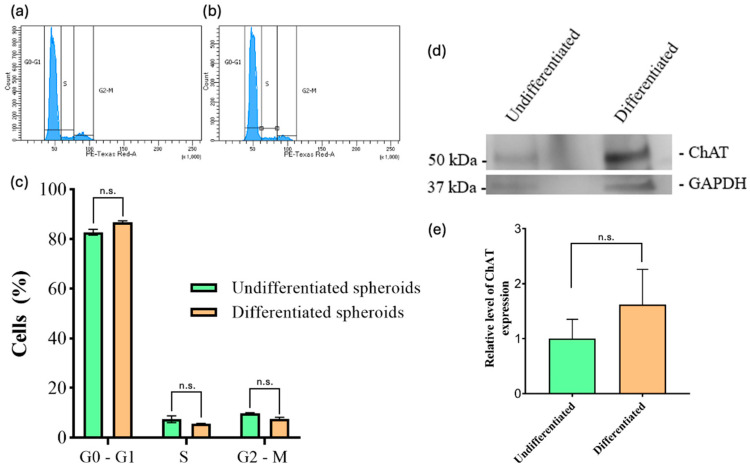
Cell cycle assay and expression of ChAT in differentiated SH-SY5Y spheroids. (**a**,**b**) Histograms of the distribution of undifferentiated (**a**) and differentiated (**b**) spheroids in growing cells labelled with propidium iodide (PI) were determined by flow cytometry. The y-axis displays the cell numbers, while the x-axis shows the PI fluorescence intensity, which correlates with the quantity of DNA per cell. (**c**) Analysis of the cell-cycle distribution of spheroids in both conditions. (**d**) Western blot analysis of ChAT protein with the loading control protein GAPDH in undifferentiated and differentiated SH-SY5Y spheroids. (**e**) Expression of ChAT levels in differentiated and undifferentiated SH-SY5Y spheroids. Data are presented as mean ± SD from three independent experiments. n.s.: not significant (*p* > 0.05).

Following a review of the protocol currently in use in our laboratory and a comparison with existing literature on the differentiation of 2D SH-SY5Y cells, we decided to implement a 22-day protocol incorporating AR, BDNF, and serum restriction as the primary variables. Figure 1 illustrates the progression of the protocol, which was initiated with a seeded density of 2000 cells per well on day 0. It also depicts the days of serum restriction and the various culture media employed to obtain SH-SY5Y spheroids differentiated into a cholinergic phenotype (ChAT+). The presence of ChAT was further validated by Western blot assay (Figure 4d), although no statistically significant difference was observed between undifferentiated and differentiated spheroids. Furthermore, a cytometry analysis was conducted to assess the impact of the differentiation protocol on the cell cycle (see Figure 4a–c). The results show no significant differences when compared to the outcomes of the corresponding controls in the percentage of cells in the G0/G1, S and G2/M phases of the cell cycle. Similarly, no differences were observed when comparing the percentages of cells in each of the G0/G1 and S phases of the cell cycle between the spheroids with differentiated and undifferentiated cells (81.7% vs. 84.4% G0/G1 phase and 4.1% vs. 4.7% S phase). However, there was a slight increase in the percentage of cells in the G2/M phase in the differentiated spheroids (10.5%) compared to the undifferentiated ones (7.4%) (Figure 4a,b).

## 3. Discussion

In this study, we established a 3D scaffold-free SH-SY5Y spheroid model capable of cholinergic differentiation under defined conditions. The model maintained structural integrity for 22 days and expressed ChAT, a key cholinergic marker, supporting its potential use in neurotoxicity screening platforms. The SH-SY5Y neuronal cell line is a commonly used human cell type among researchers, and therefore serves as an excellent model for demonstrating neuronal cell differentiation. Some methods allow the phenotypic expression of differentiation, cholinergic and glutamatergic processes to be demonstrated when SH-SY5Y cells are seeded in a monolayer in vitro [12,16]. SH-SY5Y neuroblastoma spheroids were used to simulate a complex tissue culture system with a heterogeneous microenvironment that collectively influences their growth characteristics as closely as possible.

To achieve this goal, we have conducted a detailed characterization of the system, with a particular focus on the potential alterations in spheroid growth. Once the spheroid is constituted, its 3D structure helps to overcome some limitations of traditional toxicological models, such as the absence of in vivo-like gradients, structural complexity, and cell–cell interactions.

The differentiation of SH-SY5Y cells is based on a simulated environment that occurs during in vivo neuronal development. This environment utilizes the same or similar factors and conditions as those observed in vivo. This novel operating procedure provides an accessible and reproducible method for generating homogeneous and viable spheroids. These spheroids are differentiated into mature neurons (MAP2-positive) and cholinergic neurons (ChAT-positive) from human neuroblastoma cells. The cells are taken from a heterogeneous population of adherent and suspended cells with a low passage number.

In line with the conditions of our operating procedure for the generation of dopaminergic or cholinergic neurons from SH-SY5Y cells, RA is incorporated into the cell culture medium. This is in alignment with the prevailing methodology for the induction of neuronal differentiation. RA plays a crucial role in the specification and differentiation of neural progenitors in the developing vertebrate nervous system, as well as influencing the subtype identity and neurotransmitter phenotype of maturing neurons [17,18]. Furthermore, RA has been shown to halt the progression of cells out of G0/G1 and boost PI3K/AKT activity, which is involved in the development and differentiation of neurons [19]. The regulation of serum levels in the pre-differentiation medium is also identified as a prerequisite for neuronal differentiation by several authors in their respective studies [13,20,21,22]. The initial attempts to cultivate cells in serum-free, hormone-supplemented media were conducted to elucidate the influence of serum on cell culture [23]. As demonstrated by Lu et al., the absence of serum allows for precise control over the growth factors and signaling molecules present, thereby directing cells more effectively towards a neural fate and regulating various growth factors that can promote differentiation into non-neuronal cell types [24]. The present study compares the differentiation of neuronal SH-SY5Y spheroids under normal conditions (control spheroids) and a novel operating procedure that controls RA and serum levels. As shown previously, differentiated spheroids retained circularity over time, in contrast to the control group. It is worth noting that between days 6 and 10, the control spheroids displayed a complete loss of their circularity and the morphological characteristics that are typical of this 3D model. The studies conducted by Zingales et al. and Franco-Campos et al. have demonstrated that undifferentiated spheroids derived from SH-SY5Y cells exhibit optimal characteristics on day 7 post-seeding [15,25]. However, over the following days, these spheroids gradually lose their spherical shape and SI index. In contrast, differentiated spheroids maintain their morphology and SI index over 22 days of culture (Figure 2). It is essential to maintain circularity in order to guarantee the reproducibility of experiments, establish suitable gradients (such as those of oxygen, nutrients, and metabolites), and facilitate effective cell–cell interactions. A loss of circularity in spheroids is often linked to unfavorable outcomes, including cell death, spheroid dissolution, and compromised structural integrity [26,27]. The stability of the spheroidal shape of the differentiated spheroids suggests a balanced cellular microenvironment, characterized by controlled proliferation, preserved cell–cell interactions, and consistent nutrient and oxygen diffusion. These features closely resemble those of in vivo neuronal tissue, making the model more suitable for studying neuronal differentiation and neurotoxic effects over extended periods. Compared to more complex three-dimensional (3D) neuronal models, such as induced pluripotent stem cell (iPSC)-derived spheroids and brain organoids, the SH-SY5Y spheroid system presented here offers a simple, reproducible, and scalable alternative for cholinergic differentiation. While organoid systems provide more complex cellular heterogeneity and a more intricate brain-like architecture, they often necessitate prolonged differentiation periods and complicated protocols and exhibit significant variability. In contrast, our scaffold-free model achieves consistent spheroid formation and detectable ChAT expression within 22 days, without the need for genetic manipulation or exogenous scaffolds, making it a practical platform for neurotoxicological studies.

This novel procedure also allows for the incorporation of additional factors, such as BDNF, to complete the differentiation process. BDNF plays a vital role in vivo by regulating neuronal survival, growth, and plasticity within the central nervous system. It is essential for the survival, growth, and maintenance of neurons [28,29], and has been identified as a key factor in promoting the cholinergic differentiation of SH-SY5Y neuroblastoma cells [30,31]. Specifically, it enhances the expression of choline acetyltransferase (ChAT), a pivotal enzyme in the synthesis of acetylcholine, thus supporting the acquisition of a cholinergic identify. Given the importance of the cholinergic system in cognitive processes, the inclusion of BDNF strengthens the biological relevance of our 3D differentiation model. Although no control condition without BDNF was included in this study, its incorporation into the differentiation protocol was based on previous 2D studies that demonstrated enhanced cholinergic marker expression. In our 3D spheroid model, BDNF inclusion resulted in detectable ChAT expression, supporting its relevance in promoting cholinergic differentiation under 3D conditions.

In addition to the differentiation factors employed in this operating procedure, the selection of culture medium can have a significant impact on the metabolic state and survival of neurons under diverse culture conditions. It is important to note that maintaining healthy neurons during prolonged periods of differentiation represents a significant challenge. There is a risk that some cultures may fail or become contaminated [32]. In line with the operating procedure, Neurobasal and Neurobasal Plus media are serum-free culture media that have been selected for use. These media have been observed to maintain the viability and differentiation of neuronal cells in the long term [33,34]. Furthermore, during the initial stages of differentiation, the use of highly enriched and supplemented media is of the utmost importance due to the heightened sensitivity of neuronal cells. This is a crucial consideration when conducting metabolic perturbation assays, such as the MTT redox or the cellular ATP assay. It is important to note that variations in the components of the culture medium can lead to discrepancies in the results obtained from these assays, even when testing the same cell line or substance. Femina et al. demonstrated that specific factors in the culture medium resulted in notable variability in the IC_50_ values obtained for certain drugs [35]. The key variable in the operating procedure proposed in our study is to consider both the complexity of the three-dimensional structure of the spheroids and the optimization of the culture medium (medium enriched with a balanced mixture of antioxidant enzymes, proteins, vitamins, and fatty acids) to generate ChAT-positive neurons. There are a number of ways to demonstrate the phenotypic expression of differentiation. The aim of this study was to evaluate the expression of the choline acetyltransferase (ChAT) protein in order to assess the impact of our operating procedure on neuronal differentiation development. The ChAT protein is responsible for the biosynthesis of acetylcholine and is currently the most specific indicator for monitoring the functional state of cholinergic neurons [36]. The presence of the ChAT protein is an essential indicator for identifying cholinergic neurons. This protein is, therefore, a reliable marker for designating a neuron as cholinergic [37,38]. Filograna et al. demonstrated that undifferentiated SH-SY5Y cells have a low expression of ChAT and tyrosine hydroxylase (TH) enzymes, which are, respectively, involved in acetylcholine (ACh) and dopamine (DA) synthesis [39]. The results of our study indicate that undifferentiated spheroids exhibit low ChAT expression levels, as evidenced by both immunofluorescence and Western blot assays (Figure 3 and Figure 4d). However, there was an increase in ChAT protein expression in differentiated spheroids as detected by immunofluorescence (Figure 3). However, this increase was not statistically significant when analyzed via Western blot (Figure 4d). This discrepancy may be attributed to the substantial size increase in undifferentiated spheroids, which, despite expressing ChAT at a lower percentage, may contain a large number of cells, thereby making significant changes in protein levels detectable by Western blotting.

There is a paucity of studies on differentiated SH-SY5Y cells, and there are no studies on differentiated SH-SY5Y spheroids. Nevertheless, the limited published results on differentiated neuronal cells are in line with our findings, although these studies have primarily been conducted in 2D models across different days of differentiation [9,10,12]. The process of defining a cell’s phenotype is contingent upon its proliferation rate, which is of significant consequence. The cell cycle is the focus of our study of cell proliferation rates. We take into account a number of factors, including the experimental conditions, the provenance and state of the cells, and other variables. Following mitosis, cells progress to the G1 phase of the cell cycle, during which they undergo growth and prepare for the next stage of the cell cycle. Following mitosis, some cells enter a resting phase, designated as G0. It has been observed that, in certain circumstances, some cells are able to exit this phase and subsequently re-enter the cell cycle through G1. The findings of our study demonstrate that there are no statistically significant differences between undifferentiated and differentiated cells in spheroids with regard to the cell cycle, specifically in G0/G1 and S phases (Figure 4c). This is in line with expectations, given that neurons are known to remain in the G0/G1 phase (which is characterized by cells with 2n amounts of DNA). The results of our study demonstrate that both differentiated and undifferentiated cells in spheroids are capable of entering a state of cell cycle arrest in the G1 phase (Figure 4c). The predominance of cells in the G0/G1 phase in both differentiated and undifferentiated spheroids may reflect a reduced proliferative state. However, further analysis is needed to determine whether this represents true cell cycle arrest or quiescence. Our data did not show a substantial G2/M population, but the underlying regulatory mechanisms cannot be determined from this analysis alone. The results of our study indicate that almost 84% of the total differentiated cells remained in the G0/G1 phase, which suggests that the remaining cells did not undergo differentiation under these conditions (Figure 4c). Pan et al. demonstrated that the growth of cellular spheroids in their seeding conditions can be divided into three phases: a rapid growth period, a slowdown in growth period, and a decay period [40]. In light of the undifferentiated cells, the analysis of the cell cycle and the images obtained over a period of 22 days, we can confirm that the data indicate the presence of a significant quiescent population in the G1 phase (2n or 4n) within the undifferentiated spheroids (Figure 4c). The observed DNA content profiles suggest limited cell cycle progression; however, they do not allow us to distinguish between actual arrest and other forms of reduced proliferation.

## 4. Conclusions

In conclusion, our findings present a novel operating procedure for the differentiation of SH-SY5Y cells from spheroids into cholinergic neurons (ChAT+). This novel spheroid procedure incorporates elements from the most physiologically relevant, established two-dimensional protocols, offering a valuable addition to the existing range of options. The administration of RA and brain-derived neurotrophic factor (BDNF) was found to be an effective method for promoting neuronal differentiation. The proposed operating procedure ensured the maintenance of spheroid morphology for up to 22 days, which was essential to guarantee the reproducibility and accuracy of the differentiated cholinergic neuron model. Furthermore, the expression of the cholinergic marker ChAT confirmed the successful differentiation of the cells, thus providing a three-dimensional model comprising cholinergic cells that is suitable for the study of neurotoxic effects, such as those exerted by pesticides and mycotoxins, which affect neuronal function. This model represents a novel approach to in vitro toxicity testing strategies, offering a more accurate representation of in vivo cellular behavior and providing a valuable tool for the study of cholinergic toxicity and the investigation of crucial molecular mechanisms. Furthermore, this model offers a valuable tool for implementing a profitable and scalable alternative model, thereby reducing the use of experimental animals for neurotoxicity studies. However, to gain a comprehensive understanding of the neurotoxic effects of substances, further physiological and functional studies are required.

## 5. Materials and Methods

### 5.1. Reagents

The reagent-grade chemicals and cell culture components, including DMEM/F-12. medium, Neurobasal medium (Cat #21103049), Neurobasal Plus medium (Cat #A3582901), fetal bovine serum (FBS), and trypsin/EDTA solutions, were purchased from Gibco (Waltham, MA, USA). Penicillin, streptomycin, fungizone, bovine serum albumin (BSA), phosphate-buffered saline (PBS), t-octylphenoxypolyethoxyethanol (Triton-X 100), propidium iodide (PI), 4′,6-diamidine-2′-phenylindole dihydrochloride (DAPI), trans-retinoic acid (RA) and paraformaldehyde were obtained from Sigma-Aldrich (St. Loius, MO, USA). Methanol (MeOH) and ethanol (EtOH) were obtained from Merck Life Science S.L. (Madrid, Spain). Brain-derived neurotrophic factor (BDNF) and dimethyl sulfoxide (DMSO) were obtained from Thermo Fisher Scientific (Geel, Belgium). Precast 7.5% Mini-PROTEAN TGX gels, Immuno-Blot PVDF low fluorescence membrane, Tris/glycine buffer, TBS buffer, Precision Plus Protein Western MW standard, and all other routine chemicals required for SDS-PAGE and Western blotting were purchased from Bio-Rad Laboratories (Hercules, CA, USA). The primary Monoclonal mouse MAP2 antibody (188011) was obtained from SynapticSystem (Göttingen, Germany). Polyclonal rabbit CHAT antibody (20747) and Monoclonal mouse GAPDH antibody (60004-1-Ig) were purchased from Proteintech (Chicago, IL, USA). The secondary antibodies Alexa Fluor 647^®^ Donkey Anti-Chicken (703-605-155), and Cy™3 Goat Anti-Rabbit (111-165-045) were obtained from Jackson ImmunoResearch (West Grove, PA, USA).

### 5.2. SH-SY5Y Cell Culture

SH-SY5Y human neuroblastoma cells were procured from the American Type Culture Collection (ATCC CRL-2266). Cells were maintained in DMEM/F-12 medium supplemented with 10% FBS, 100 U/mL penicillin, 100 mg/mL streptomycin, and 0.2% fungizone. The incubation conditions were maintained at a pH of 7.4, with 5% CO2 at 37 °C and 95% air atmosphere. The culture medium was changed every three days, and to ensure genetic homogeneity, cells were commonly subcultured twice a week, with a maximum of 25 subpassages. Mycoplasma absence was regularly verified through the MycoAlert™ PLUS Mycoplasma Kit (Rockland, ME, USA). Approximately 2 × 10^3^ SH-SY5Y cells in mL were added to each well of ultra-low attachment (ULA) round-bottomed 96-well plates (Corning^®^, Corning NY, USA) with 10% FBS medium, as described previously by Zingales et al., to form spheroids in the following days [15].

### 5.3. SH-SY5Y Spheroid Differentiation

To initiate highly reproducible SH-SY5Y spheroids differentiated into a cholinergic phenotype, on day 2, the pre-differentiation DMEM/F-12 medium was aspirated, and the cells received DMEM/F-12 medium supplemented with 5% of FBS and 10 µM RA. From this day forward, the medium was changed daily (until day 6 after seeding) in order to decrease the percentage of FBS until it reaches 1%. On day 7, the medium was changed to “differentiation medium (a)” consisting of Neurobasal Plus medium supplemented with 10 µM RA and 50 ng/mL BDNF. The medium was renewed on days 10 and 13 post seeding. On day 16, the medium was changed to “differentiation medium (b)” consisting of Neurobasal medium supplemented with 10 µM RA and 50 ng/mL BDNF, which was renewed every 3 days until day 22 post seeding, when the differentiated spheroids were considered ready for use.

To guarantee the reliability of the results, it is essential to verify that the spheroids are consistently uniform in size and shape. Therefore, morphological parameters such as solidity and sphericity index (SI) were assessed. The parameters were evaluated for spheroids formed under both the standard DMEM/F12 medium and the differentiation protocol established in this work. Bright-field images were captured immediately after cell seeding and subsequently every 3 days over a 22-day period. Spheroids are considered to be of a regular shape if their solidity values exceed 0.90. Similarly, Zanoni et al. defined spheroids as spherical if their SI was equal to or greater than 0.90 [41] The SI was calculated using the equation below:

$$SI=\frac{\pi \sqrt{4A/\pi }}{\pi d}$$

where A and d are the area and diameter of the spheroid, respectively. Image analysis was performed using a Zeiss Primo Vert inverted microscope equipped with an Axiocam 208 color camera (Zeiss Microscopy, Jena, Germany) at 10× magnification and ImageJ software (version 1.53t; NIH, Bethesda, MD, USA) to obtain the SI index of each spheroid.

### 5.4. Cell Cycle Analysis by Flow Cytometry

Vindeløv’s PI staining solution was utilized to analyze the cell cycle, adhering to the protocol described by Vindeløv [42]. PI, a fluorescent dye, binds to double-stranded DNA, allowing for the precise measurement of cellular DNA content via flow cytometry. Briefly, 24 spheroids per condition were immediately placed in an Eppendorf tube and disaggregated with trypsin for 5 min at 37 °C. Suspended cells were collected, washed once in 1× PBS buffer, and centrifuged at 1200 rpm for 5 min. The supernatant was discarded, and the pellet was resuspended in 100 μL of 1× PBS buffer. The cells were fixed with cold 70% ethanol for 30 min and washed with PBS. Cells were transferred to a 5 mL flow cytometry tube and incubated with 1 μL of staining solution containing 40 µg/mL RNase, 0.1% Triton X-100, 10 nM Tris, 50 µg/mL of PI and 10 nm NaCL in PBS. Samples were analyzed on a Cytoflex flow cytometer (Beckman Coulter, San Diego, CA, USA). Three independent experiments were performed, and 10,000 cells dissociated from spheroids were taken and analyzed per sample using a BD LSR-Fortessa flow cytometer (BD Biosciences, Franklin Lakes, NJ, USA).

### 5.5. Immunofluorescence Detection

Indirect immunofluorescence was employed to analyze different markers, including neuronal (MAP2 (microtubule-associated protein 2)) and cholinergic (ChAT (acetylcholinesterase)) markers. For all experiments, primary and secondary antibodies were prepared as follows: MAP2 (1:250), ChAT (1:200), Cy™3 (1:300), and Alexa Fluor 647^®^ (1:300). Once the differentiation process was completed, PBS was used to wash the differentiated spheroids and controls, followed by fixation with 4% formaldehyde for one hour at room temperature. After the incubation period, the medium was discarded, and spheroids were washed three times with PBS while being gently agitated at room temperature. Subsequently, the spheroids were stored at 4 °C. After 24 h, they were permeabilized and blocked overnight using 1 mL of 0.3% Triton X-100 in PBS, together with 1 mL of a blocking solution containing 20% horse serum and 2% BSA in PBS. Following a 24 h period, spheroids were washed with PBS and incubated overnight at 4 °C with primary antibodies (MAP2 and ChAT) diluted in PBS, 2% BSA, and 0.2% Triton X-100. On the following day, after several washes with PBS, the spheroids were incubated with secondary antibodies (Cy™3 and Alexa Fluor 647 diluted in PBS, 2% BSA and 0.2% Triton X-100) and DAPI (1:1000) for a period of three hours at room temperature. The spheroids were subsequently placed in an 8-well glass-bottom μ-slide (ibidi GmbH, Gräfelfing, Germany). Images were captured using a confocal fluorescence microscope (Fluoview FV1000; Olympus, Tokyo, Japan), maintaining consistent acquisition settings. Z-stacks were collected along the spheroid periphery using automated microscopy, with images acquired at a resolution of 1024 × 1024 pixels and a 1 µm optical section thickness.

### 5.6. Western Blot Assay

Western blot analysis was conducted on proteins extracted from undifferentiated and differentiated SH-SY5Y spheroids following the completion of the differentiation protocol. Thirty spheroids were selected from each condition, the cells were lysed, and the proteins were extracted in 200 µL of RIPA buffer with a protease and phosphatase inhibitor cocktail (Santa Cruz Biotechnology, Santa Cruz, CA, USA). The samples were then centrifuged at 12,000 rpm and 4 °C for 15 min to collect the cellular proteins in the supernatants. The protein concentrations were subsequently quantified using a modified Bradford assay (BioRad Laboratories, Madrid, Spain) with the Cydex system [43] and the samples prepared with 25 µg of protein each. The samples were separated by 7.5% sodium dodecyl sulfate-polyacrylamide gel electrophoresis (SDS-PAGE) under reducing conditions and transferred to a polyvinylidene difluoride (PVDF) membrane (BioRad Laboratories), ensuring that the protein amounts in each sample were equal. The membranes were blocked for one hour at room temperature in 5% (*w*/*v*) non-fat milk in buffer TBS-Tween. They were incubated overnight at 4 °C with rabbit anti-ChAT (1:400) and mouse anti-GAPDH (1:500) in blocking buffer. Subsequently, the blots were washed three times with TBS-Tween buffer, blocked, and incubated with mouse and rabbit, respectively—HRP-conjugated secondary antibodies (1:2500)—for 2 h at room temperature. The protein bands were visualized using the enhanced chemiluminescence (ECL) method (ThermoFisher Scientific, Cambridge, MA, USA), and quantified using ImageJ software (version 1.53t; NIH, Bethesda, MD, USA)

### 5.7. Statistical Analysis

Statistical analysis was performed using GraphPad Prism version 8 (GraphPad Software, San Diego, CA, USA). Data are presented as mean values ± standard error of the mean (SEM) from multiple independent experiments. Comparisons between groups were assessed using one-way analysis of variance (ANOVA), followed by Tukey’s Honestly Significant Difference (HSD) post hoc test. *p* ≤ 0.05 was considered statistically significant.

## Data Availability

The original contributions presented in this study are included in this article. Further inquiries can be directed to the corresponding author.

## Abbreviations

The following abbreviations are used in this manuscript:

DOAJ	Directory of Open Access Journals
TLA	Three-letter Acronym
LD	Linear Dichroism
